# Supplementary material for: Modelling Skylarks (Alauda arvensis) to Predict Impacts of Changes in Land Management and Policy: Development and Testing of an Agent-Based Model
Source: PLoS One. 2013 Jun 6;8(6):e65803. doi: 10.1371/journal.pone.0065803 (PMC3675089; doi:10.1371/journal.pone.0065803)
Supplement: Supporting Information S4 — The skylark ODdox as a zipped archive. (ZIP) [file pone.0065803.s004.zip › Skylark_ODdox/class_user_defined_farm.html]

ALMaSS Skylark ODdox: UserDefinedFarm Class Reference


ALMaSS Skylark ODdox
 2.0


- Main Page
- Related Pages
- Classes
- Files

- Class List
- Class Index
- Class Hierarchy
- Class Members

Public Member Functions |
Protected Member Functions

UserDefinedFarm Class Reference

A farm that can have its rotation defined by the user at runtime.
More...

`#include <farm.h>`

List of all members.

|  |  |
| --- | --- |
| Public Member Functions | |
|  | UserDefinedFarm (const char \*fname) |
| Public Member Functions inherited from Farm | |
| void | AddField (LE \*a\_newfield) |
|  | Adds a field to a farm. |
| void | AddNewEvent (TTypesOfVegetation a\_event, long a\_date, LE \*a\_field, int a\_todo, long a\_num, bool a\_lock, int a\_start, bool a\_first\_year, TTypesOfVegetation a\_crop) |
|  | Adds an event to the event queue for a farm. |
| virtual bool | AutumnHarrow (LE \*a\_field, double a\_user, int a\_days) |
|  | Carry out a harrow event in the autumn on a\_field. |
| virtual bool | AutumnPlough (LE \*a\_field, double a\_user, int a\_days) |
|  | Carry out a ploughing event in the autumn on a\_field. |
| virtual bool | AutumnRoll (LE \*a\_field, double a\_user, int a\_days) |
|  | Carry out a roll event in the autumn on a\_field. |
| virtual bool | AutumnSow (LE \*a\_field, double a\_user, int a\_days) |
|  | Carry out a sowing event in the autumn on a\_field. |
| virtual bool | BurnStrawStubble (LE \*a\_field, double a\_user, int a\_days) |
|  | Burn stubble on a\_field. |
| virtual bool | CattleIsOut (LE \*a\_field, double a\_user, int a\_days, int a\_max) |
|  | Generate a 'cattle\_out' event for every day the cattle are on a\_field. |
| virtual bool | CattleIsOutLow (LE \*a\_field, double a\_user, int a\_days, int a\_max) |
|  | Generate a 'cattle\_out\_low' event for every day the cattle are on a\_field. |
| virtual bool | CattleOut (LE \*a\_field, double a\_user, int a\_days) |
|  | Start a grazing event on a\_field today. |
| virtual bool | CattleOutLowGrazing (LE \*a\_field, double a\_user, int a\_days) |
|  | Start a extensive grazing event on a\_field today. |
| virtual bool | CutToHay (LE \*a\_field, double a\_user, int a\_days) |
|  | Carry out hay cutting on a\_field. |
| virtual bool | CutToSilage (LE \*a\_field, double a\_user, int a\_days) |
|  | Cut vegetation for silage on a\_field. |
| virtual bool | CutWeeds (LE \*a\_field, double a\_user, int a\_days) |
|  | Carry out weed topping on a\_field. |
| virtual bool | DeepPlough (LE \*a\_field, double a\_user, int a\_days) |
|  | Carry out a deep ploughing event on a\_field. |
| bool | DoIt (int a\_probability) |
|  | Return chance out of 100. |
| virtual bool | FA\_AmmoniumSulphate (LE \*a\_field, double a\_user, int a\_days) |
|  | Apply ammonium supahte to a\_field owned by an stock farmer. |
| virtual bool | FA\_GreenManure (LE \*a\_field, double a\_user, int a\_days) |
|  | Spread green manure on a\_field owned by an stock farmer. |
| virtual bool | FA\_Manure (LE \*a\_field, double a\_user, int a\_days) |
|  | Spread manure on a\_field owned by an stock farmer. |
| virtual bool | FA\_NPK (LE \*a\_field, double a\_user, int a\_days) |
|  | Apply NPK fertilizer to a\_field owned by an stock farmer. |
| virtual bool | FA\_PK (LE \*a\_field, double a\_user, int a\_days) |
|  | Apply PK fertilizer to a\_field owned by an stock farmer. |
| virtual bool | FA\_Sludge (LE \*a\_field, double a\_user, int a\_days) |
|  | Spread sewege sludge on a\_field owned by an stock farmer. |
| virtual bool | FA\_Slurry (LE \*a\_field, double a\_user, int a\_days) |
|  | Spready slurry on a\_field owned by an stock farmer. |
|  | Farm (void) |
|  | Farm constructor - creates an instance of each possible crop type. |
| virtual bool | FP\_GreenManure (LE \*a\_field, double a\_user, int a\_days) |
|  | Spread green manure on a\_field owned by an arable farmer. |
| virtual bool | FP\_LiquidNH3 (LE \*a\_field, double a\_user, int a\_days) |
|  | Apply liquid ammonia fertilizer to a\_field owned by an arable farmer. |
| virtual bool | FP\_ManganeseSulphate (LE \*a\_field, double a\_user, int a\_days) |
|  | Apply Manganse Sulphate to a\_field owned by an arable farmer. |
| virtual bool | FP\_Manure (LE \*a\_field, double a\_user, int a\_days) |
|  | Spread manure on a\_field owned by an arable farmer. |
| virtual bool | FP\_NPK (LE \*a\_field, double a\_user, int a\_days) |
|  | Apply NPK fertilizer, on a\_field owned by an arable farmer. |
| virtual bool | FP\_NPKS (LE \*a\_field, double a\_user, int a\_days) |
|  | Apply NPKS fertilizer, on a\_field owned by an arable farmer. |
| virtual bool | FP\_PK (LE \*a\_field, double a\_user, int a\_days) |
|  | Apply PK fertilizer, on a\_field owned by an arable farmer. |
| virtual bool | FP\_Sludge (LE \*a\_field, double a\_user, int a\_days) |
|  | Spread sewege on a\_field owned by an arable farmer. |
| virtual bool | FP\_Slurry (LE \*a\_field, double a\_user, int a\_days) |
|  | Apply slurry to a\_field owned by an arable farmer. |
| virtual bool | FungicideTreat (LE \*a\_field, double a\_user, int a\_days) |
|  | Apply fungicide to a\_field. |
| int | GetArea (void) |
|  | Returns the area of arable fields owned by that farm. |
| int | GetFarmNumber (void) |
| int | GetIntensity (void) |
| TTypesOfFarm | GetType (void) |
| virtual bool | Glyphosate (LE \*a\_field, double a\_user, int a\_days) |
| virtual bool | GrowthRegulator (LE \*a\_field, double a\_user, int a\_days) |
|  | Apply growth regulator to a\_field. |
| virtual bool | Harvest (LE \*a\_field, double a\_user, int a\_days) |
|  | Carry out a harvest on a\_field. |
| virtual bool | HayBailing (LE \*a\_field, double a\_user, int a\_days) |
|  | Carry out hay bailing on a\_field. |
| virtual bool | HayTurning (LE \*a\_field, double a\_user, int a\_days) |
|  | Carry out hay turning on a\_field. |
| virtual bool | HerbicideTreat (LE \*a\_field, double a\_user, int a\_days) |
|  | Apply herbicide to a\_field. |
| virtual bool | HillingUp (LE \*a\_field, double a\_user, int a\_days) |
|  | Do hilling up on a\_field, probably of potatoes. |
| virtual bool | InsecticideTreat (LE \*a\_field, double a\_user, int a\_days) |
|  | Apply insecticide to a\_field. |
| bool | IsStockFarmer (void) |
| virtual void | MakeStockFarmer (void) |
| virtual void | Management (void) |
|  | Starts the main management loop for the farm and performs some error checking. |
| virtual bool | Molluscicide (LE \*a\_field, double a\_user, int a\_days) |
|  | Apply molluscidie to a\_field. |
| virtual bool | PigsAreOut (LE \*a\_field, double a\_user, int a\_days) |
|  | Start a pig grazing event on a\_field today or soon. |
| virtual bool | PigsAreOutForced (LE \*a\_field, double a\_user, int a\_days) |
|  | Start a pig grazing event on a\_field today - no exceptions. |
| virtual bool | PigsOut (LE \*a\_field, double a\_user, int a\_days) |
|  | Generate a 'pigs\_out' event for every day the cattle are on a\_field. |
| virtual bool | ProductApplication0 (LE \*a\_field, double a\_user, int a\_days) |
|  | Apply test pesticide to a\_field. |
| virtual bool | ProductApplication1 (LE \*a\_field, double a\_user, int a\_days) |
|  | Apply test pesticide to a\_field. |
| void | RemoveField (LE \*a\_field) |
|  | Removes a field to a farm. |
| virtual bool | RowCultivation (LE \*a\_field, double a\_user, int a\_days) |
|  | Carry out a harrowing between crop rows on a\_field. |
| void | SetFarmNumber (int a\_farm\_num) |
| virtual bool | SleepAllDay (LE \*a\_field, double a\_user, int a\_days) |
|  | Nothing to to today on a\_field. |
| virtual bool | SpringHarrow (LE \*a\_field, double a\_user, int a\_days) |
|  | Carry out a harrow event in the spring on a\_field. |
| virtual bool | SpringPlough (LE \*a\_field, double a\_user, int a\_days) |
|  | Carry out a ploughing event in the spring on a\_field. |
| virtual bool | SpringRoll (LE \*a\_field, double a\_user, int a\_days) |
|  | Carry out a roll event in the spring on a\_field. |
| virtual bool | SpringSow (LE \*a\_field, double a\_user, int a\_days) |
|  | Carry out a sowing event in the spring on a\_field. |
| virtual bool | StrawChopping (LE \*a\_field, double a\_user, int a\_days) |
|  | Carry out straw chopping on a\_field. |
| virtual bool | Strigling (LE \*a\_field, double a\_user, int a\_days) |
|  | Carry out a mechanical weeding on a\_field. |
| virtual bool | StriglingSow (LE \*a\_field, double a\_user, int a\_days) |
|  | Carry out a mechanical weeding followed by sowing on a\_field. |
| virtual bool | StubbleHarrowing (LE \*a\_field, double a\_user, int a\_days) |
|  | Carry out stubble harrowing on a\_field. |
| virtual bool | Swathing (LE \*a\_field, double a\_user, int a\_days) |
|  | Cut the crop on a\_field and leave it lying (probably rape) |
| virtual bool | SynInsecticideTreat (LE \*a\_field, double a\_user, int a\_days) |
|  | Apply special insecticide to a\_field. |
| TTypesOfVegetation | TranslateCropCodes (std::string &str) |
| virtual bool | Trial\_Control (LE \*a\_field, double a\_user, int a\_days) |
|  | Special pesticide trial functionality. |
| virtual bool | Trial\_PesticideTreat (LE \*a\_field, double a\_user, int a\_days) |
|  | Special pesticide trial functionality. |
| virtual bool | Trial\_PesticideTreat\_GS (LE \*a\_field, double a\_user, int a\_days) |
|  | Special pesticide trial functionality. |
| virtual bool | Trial\_ToxicControl (LE \*a\_field, double a\_user, int a\_days) |
|  | Special pesticide trial functionality. |
| virtual bool | Water (LE \*a\_field, double a\_user, int a\_days) |
|  | Carry out a watering on a\_field. |
| virtual bool | WinterPlough (LE \*a\_field, double a\_user, int a\_days) |
|  | Carry out a ploughing event in the winter on a\_field. |
| virtual | ~Farm (void) |
|  | Farm destructor - deletes all crop instances and empties event queues. |

|  |  |
| --- | --- |
| Protected Member Functions | |
| void | AssignPermanentCrop (TTypesOfVegetation tov, int pct) |
|  | Used to assign a permanent crop to an otherwise rotational field polygon. |
| virtual void | InitiateManagement (void) |
|  | Kicks off the farm's management. |
| int | InvIntPartition (vector< tpct > \*items, int target) |
|  | Finds all possible sums of the integers in the items array. |
| Protected Member Functions inherited from Farm | |
| void | CheckRotationManagementLoop (FarmEvent \*ev) |
| virtual int | GetFirstCropIndex (TTypesOfLandscapeElement a\_type) |
|  | Gets the first crop for the farm. |
| int | GetFirstDate (TTypesOfVegetation a\_tov) |
|  | Gets the start date for a crop type. |
| virtual int | GetNextCropIndex (int a\_rot\_index) |
|  | Returns the next crop in the rotation. |
| int | GetNextCropStartDate (LE \*a\_field, TTypesOfVegetation &a\_curr\_veg) |
|  | Returns the start date of the next crop in the rotation. |
| void | HandleEvents (void) |
|  | If there are events to carry out do this, and perhaps start a new crop. |
| bool | LeSwitch (FarmEvent \*ev) |
|  | Call do function for any crop with an outstanding event. Signal if the crop has terminated. |
| void | ReadRotation (std::string fname) |
|  | Reads a rotation file into the rotation. |

|  |  |
| --- | --- |
| Additional Inherited Members | |
| Protected Attributes inherited from Farm | |
| AgroChemIndustryCereal \* | m\_agrochemindustrycereal |
| Carrots \* | m\_carrots |
| CloverGrassGrazed1 \* | m\_CGG1 |
| CloverGrassGrazed2 \* | m\_CGG2 |
| int | m\_farm\_num |
| TTypesOfFarm | m\_farmtype |
| FieldPeas \* | m\_fieldpeas |
| FieldPeasStrigling \* | m\_fieldpeasstrigling |
| vector< LE \* > | m\_fields |
| Fodderbeet \* | m\_fodderbeet |
| FodderGrass \* | m\_foddergrass |
| int | m\_intensity |
| Maize \* | m\_maize |
| MaizeSilage \* | m\_maizesilage |
| MaizeStrigling \* | m\_maizestrigling |
| Oats \* | m\_oats |
| OBarleyPeaCloverGrass \* | m\_OBarleyPCG |
| OCarrots \* | m\_ocarrots |
| OCloverGrassGrazed1 \* | m\_OCGG1 |
| OCloverGrassGrazed2 \* | m\_OCGG2 |
| OCloverGrassSilage1 \* | m\_OCGS1 |
| OFieldPeas \* | m\_ofieldpeas |
| OFieldPeasSilage \* | m\_ofieldpeassilage |
| OFirstYearDanger \* | m\_ofirstyeardanger |
| OGrazingPigs \* | m\_ograzingpigs |
| OMaizeSilage \* | m\_omaizesilage |
| OOats \* | m\_ooats |
| OPermanentGrassGrazed \* | m\_opermgrassgrazed |
| OPotatoes \* | m\_opotatoes |
| OSBarleySilage \* | m\_osbarleysilage |
| OSpringBarley \* | m\_ospringbarley |
| OSpringBarleyExt \* | m\_ospringbarleyext |
| OSpringBarleyPigs \* | m\_ospringbarleypigs |
| OWinterBarley \* | m\_owinterbarley |
| OWinterBarleyExt \* | m\_owinterbarleyext |
| OWinterRape \* | m\_owinterrape |
| OWinterRye \* | m\_owinterrye |
| OWinterWheatUndersown \* | m\_owinterwheatundersown |
| OWinterWheatUndersownExt \* | m\_owinterwheatundersownext |
| PermanentSetAside \* | m\_permanentsetaside |
| vector< PermCropData > | m\_PermCrops |
| PermanentGrassGrazed \* | m\_permgrassgrazed |
| PermanentGrassLowYield \* | m\_permgrasslowyield |
| PermanentGrassTussocky \* | m\_permgrasstussocky |
| Potatoes \* | m\_potatoes |
| PotatoesIndustry \* | m\_potatoesindustry |
| LowPriority< FarmEvent \* > | m\_queue |
| vector< TTypesOfVegetation > | m\_rotation |
| int | m\_rotation\_sync\_index |
| SpringBarleyCloverGrass \* | m\_sbarleyclovergrass |
| SeedGrass1 \* | m\_seedgrass1 |
| SeedGrass2 \* | m\_seedgrass2 |
| SetAside \* | m\_setaside |
| SpringBarley \* | m\_springbarley |
| SpringBarleyCloverGrassStrigling \* | m\_springbarleyclovergrassstrigling |
| SpringBarleyPeaCloverGrassStrigling \* | m\_springbarleypeaclovergrassstrigling |
| SpringBarleyPTreatment \* | m\_springbarleyptreatment |
| SpringBarleySeed \* | m\_springbarleyseed |
| SpringBarleySilage \* | m\_springbarleysilage |
| SpringBarleySKManagement \* | m\_springbarleyskmanagement |
| SpringBarleyStrigling \* | m\_springbarleystrigling |
| SpringBarleyStriglingCulm \* | m\_springbarleystriglingculm |
| SpringBarleyStriglingSingle \* | m\_springbarleystriglingsingle |
| SpringRape \* | m\_springrape |
| bool | m\_stockfarmer |
| Triticale \* | m\_triticale |
| WinterBarley \* | m\_winterbarley |
| WinterBarleyStrigling \* | m\_winterbarleystrigling |
| WinterRape \* | m\_winterrape |
| WinterRapeStrigling \* | m\_winterrapestrigling |
| WinterRye \* | m\_winterrye |
| WinterRyeStrigling \* | m\_winterryestrigling |
| WinterWheat \* | m\_winterwheat |
| WinterWheatStrigling \* | m\_winterwheatstrigling |
| WinterWheatStriglingCulm \* | m\_winterwheatstriglingculm |
| WinterWheatStriglingSingle \* | m\_winterwheatstriglingsingle |
| WWheatPControl \* | m\_wwheatpcontrol |
| WWheatPToxicControl \* | m\_wwheatptoxiccontrol |
| WWheatPTreatment \* | m\_wwheatptreatment |
| YoungForestCrop \* | m\_youngforest |

---

## Detailed Description

A farm that can have its rotation defined by the user at runtime.

The rotation is controlled by a file with the format: N  
Crop  
Crop  
Crop...  
..........where N is the number of entries and "Crop" is replaced by the crop name for each entry in the rotation. There are 16 Userdefined farm types available numbered 1 to 16, but 2->16 have been excluded from the documentation because they are identical to this class in all but name.

---

## Constructor & Destructor Documentation

|  |  |  |  |  |  |
| --- | --- | --- | --- | --- | --- |
| UserDefinedFarm::UserDefinedFarm | ( | const char \* | *fname* | ) |  |

User defined farms derived from this class have greater functionality and greater flexibility than those defined from Farm. There are three areas of added functionality:  
1) The ability to define whether the farm is arable or stock  
2) The ability to define farm intensity   
3) The capability of defining permanent crops (e.g. permanent grass or silage maize). This means that whatever field is assigned this crop in the first year will retain it subsequently. Further functionality here is that the ideal crop area can also be specified. If so then the farmer will identify the field closest to that area and assign the crop to that.

References g\_msg, Farm::m\_PermCrops, Farm::m\_rotation, Farm::m\_stockfarmer, PermCropData::Pct, PermCropData::Tov, Farm::TranslateCropCodes(), MapErrorMsg::Warn(), and WARN\_FILE.

: Farm() // Base class for the real ones

{

ifstream ifile;

ifile.open(fname,ios::in);

if (!ifile.is\_open()) {

g\_msg->Warn( WARN\_FILE, " Unable to open file ", fname );

exit( 1 );

}

int input, intensity;

// First entry - arable/stock

// Second entry - farm intensity. Zero = default, 1 extensive, 2 very extensive

ifile >> input >> intensity;

if (input==1) m\_stockfarmer = true; else m\_stockfarmer = false;

// Thrid entry - no permanent crops

int permcrops;

std::string cropref ="";

ifile >> permcrops;

// The problem here is that at the time when we reach this code there are no fields attached to the farm - so we need

// to store this information - hence the vector of the vector of PermCropData

PermCropData pcd;

for (int i=0; i<permcrops; i++) {

int pct;

ifile >> cropref;

TTypesOfVegetation tov = TranslateCropCodes( cropref );

ifile >> pct; // 0-100%

pcd.Pct=pct;

pcd.Tov=tov;

m\_PermCrops.push\_back(pcd);

}

// 4th entry

int nocrops;

ifile >> nocrops;

m\_rotation.resize( nocrops );

for ( int i = 0; i < nocrops; i++ ) {

ifile >> cropref;

TTypesOfVegetation tov = TranslateCropCodes( cropref );

m\_rotation[ i ] = tov;

}

ifile.close();

}

---

## Member Function Documentation

|  |  |  |  |
| --- | --- | --- | --- |
| void UserDefinedFarm::AssignPermanentCrop | ( | TTypesOfVegetation | *tov*, |
|  |  | int | *pct* |
|  | ) |  |  |

protected

Used to assign a permanent crop to an otherwise rotational field polygon.

References CompPcts(), Farm::GetArea(), tpct::index, InvIntPartition(), Farm::m\_fields, tpct::pct, random(), and tov\_Undefined.

{

// Assumes that m\_fields has all the fields already in it, and those that are occupied by permanent tole types

// are denoted by -2 or -4 in rotindex, -1 is unassigned - can't use enum because positive values have other meanings

// First get the areas of all fields and then convert these to percentages

tpct a\_tpct;

vector<LE\*> fields\_cpy;

fields\_cpy.resize(m\_fields.size());

copy(m\_fields.begin(), m\_fields.end(), fields\_cpy.begin());

vector<tpct> pcts;

double area = 0.0;

int sz = (int) fields\_cpy.size();

for (int i=sz-1; i>=0; i--) {

if ( fields\_cpy[i]->GetRotIndex()<-1) {

if (fields\_cpy[i]->GetRotIndex()==-4) area += fields\_cpy[i]->GetArea();

fields\_cpy.erase(fields\_cpy.begin()+i);

}

else area += fields\_cpy[i]->GetArea();

}

// Here we can take action if we only have a single field (could extend this if it was thought worth it)

int fnos = (int) fields\_cpy.size();

if (fnos<1) return; // No fields to allocate, jump out

else if (fnos<2) {

// Use the pct as the chance that our single field is assigned

if (random(100)>=pct) return;

else {

// Add field by default

fields\_cpy[0]->SetRotIndex(-4);

// Assign the crop

fields\_cpy[0]->SetVegType(tov, tov\_Undefined);

return;

}

}

for (int i=0; i<fnos; i++) {

a\_tpct.pct = (int) floor(0.5+((fields\_cpy[i]->GetArea()/area)\*100));

a\_tpct.index = i;

pcts.push\_back(a\_tpct);

}

// We need to look for combinations of fields that are close to our target.

// First we can sort the array and ignore any that are greater than pct

sort (pcts.begin(), pcts.end(), CompPcts); // results in ordering on increasing pct

// now remove all those with pct>target+10% to save loads of time in the inverse integer partition

int index=-1;

int ind=0;

int apct=pct+10; // Lets assume we have a tolerance of 10%

while ((index==-1) && (ind< (int)pcts.size())) {

if (pcts[ind++].pct>apct) index=ind;

}

if (index!=-1) pcts.erase(pcts.begin()+index,pcts.end());

// Now find the best combination of fields to get close to our target & set them

int bits = InvIntPartition(&pcts,pct);

int mask=1;

ind = 0;

int used=0;

double pctused = 0.0;

double check = 0.0;

for (int h=0; h< (int)pcts.size(); h++) check+=fields\_cpy[pcts[h].index]->GetArea();

while (bits>0) {

if ((bits & mask) > 0) {

// Set the field as non-rotating

fields\_cpy[pcts[ind].index]->SetRotIndex(-4);

// Assign the crop

fields\_cpy[pcts[ind].index]->SetVegType(tov, tov\_Undefined);

used += (int) fields\_cpy[pcts[ind].index]->GetArea();

pctused = used/area;

bits -= mask;

}

mask = mask << 1;

ind++;

}

}

|  |  |  |  |  |  |
| --- | --- | --- | --- | --- | --- |
| void UserDefinedFarm::InitiateManagement | ( | void |  | ) |  |

protectedvirtual

Kicks off the farm's management.

Reimplemented from Farm.

Reimplemented in UserDefinedFarm36.

References Farm::InitiateManagement().

{

// First we need to assign the permanent crops if any.

if (m\_PermCrops.size()>0) {

// We have something to do

for (int i=0; i<(int)m\_PermCrops.size(); i++) {

AssignPermanentCrop(m\_PermCrops[i].Tov, m\_PermCrops[i].Pct);

}

}

Farm::InitiateManagement();

}

|  |  |  |  |
| --- | --- | --- | --- |
| int UserDefinedFarm::InvIntPartition | ( | vector< tpct > \* | *items*, |
|  |  | int | *target* |
|  | ) |  |  |

protected

Finds all possible sums of the integers in the items array.

References g\_msg, Farm::GetFarmNumber(), and MapErrorMsg::Warn().

Referenced by AssignPermanentCrop().

{

//Figure out how many bitmasks we need...

//4 bits have a maximum value of 15, so we need 15 masks.

//Calculated as:

// (2 ^ ItemCount) - 1

int sz = (int) items->size();

if (sz>63) {

g\_msg->Warn("Too many potential fields in UserDefinedFarm::InvIntPartition: ",sz);

g\_msg->Warn("Farm Number: ",GetFarmNumber());

exit(1);

}

long int calcs = (1 << sz);

//Spit out the corresponding calculation for each bitmask

int sum;

int found = 0;

int diff = 100;

for (long int i=1; i<calcs; i++) {

//Get the items from our array that correspond to

//the on bits in our mask

sum = 0;

int mask = 1;

for (int bit=0; bit<sz; bit++) {

if ((i & mask) > 0) {

sum+=(\*items)[bit].pct;

}

mask = mask << 1;

}

if (abs(sum-target) < diff ){

found = i;

diff = abs(sum-target);

}

}

return found;

}

---

The documentation for this class was generated from the following files:

- farm.h
- farm.cpp


- UserDefinedFarm
- Generated on Thu Jan 10 2013 13:15:38 for ALMaSS Skylark ODdox by
   1.8.1.1
